# Supplementary material for: β subunits of GABAA receptors form proton-gated chloride channels: Insights into the molecular basis
Source: Commun Biol. 2022 Aug 3;5:784. doi: 10.1038/s42003-022-03720-2 (PMC9349252; doi:10.1038/s42003-022-03720-2)
Supplement: Supplementary file 2 — Supplementary Information [file 42003_2022_3720_MOESM2_ESM.pdf]

β subunits of $GABA_A$ receptors form proton-gated chloride channels: Insights into the molecular basis

Aleksandra Garifulina[1]*, Theres Friesacher[1], Marco Stadler[1], Eva-Maria Zangerl-Plessl[1], Margot Ernst[2], Anna Stary-Weinzinger[1], Anita Willam[1], Steffen Hering[1]*

[1] Division of Pharmacology and Toxicology, Department of Pharmaceutical Sciences, University of Vienna, A-1090 Vienna, Austria

[2] Department of Pathobiology of the Nervous System, Medical University of Vienna, A-1090 Vienna, Austria

*Corresponding authors: Aleksandra Garifulina (aleksandra.garifulina@univie.ac.at), Steffen Hering (steffen.hering@univie.ac.at)

Supplementary information

Supplementary Figures

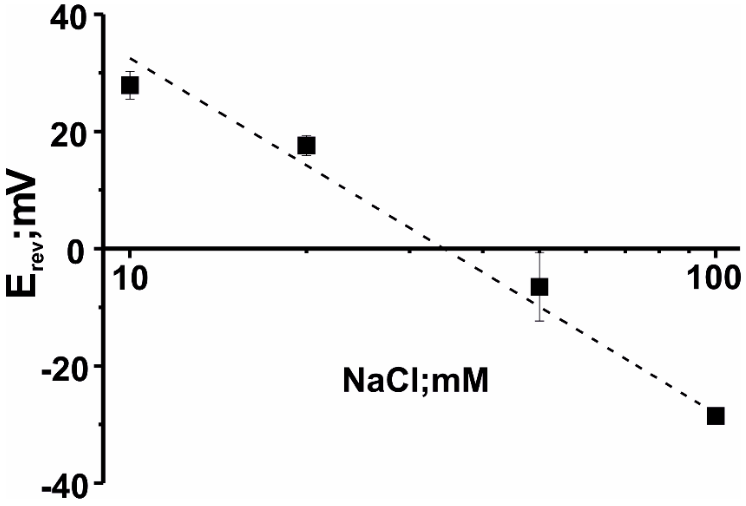

Supplementary Figure 1. Homomeric β3 (rat) $GABA_A$ receptors are selective for chloride. Dependence of the reversal potential (E_{rev}) of $I_{H(β3)}$ on the extracellular NaCl concentrations. The regression line with the negative slope of about -58 mV corresponds to selective permission of chloride ions. See also similar data for β1 $GABA_AR$ in [1].

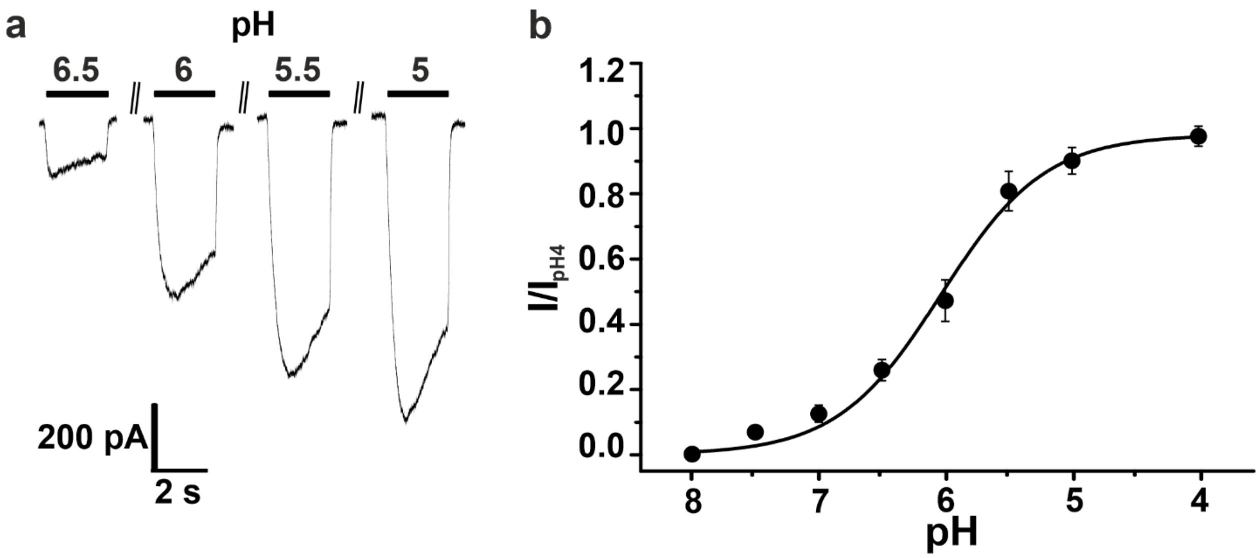

Supplementary Figure 2. Proton-induced currents ($I_{H(\beta)}$) through homomeric (human) GABA$_A$ β3 receptor expressed in CHO cells.

a Representative $I_{H(\beta)}$ through (human) β3 subunit homomers elicited by rapidly changing the pH from 7.4 to the indicated values. **b** Normalized pH-response curve of $I_{H(\beta)}$ in CHO cells expressing β3 GABA$_A$R (pH_{50} = 6.03 ± 0.19 and n_H =1.06 ± 0.33). The data are presented as mean values ±SEM, n=14.

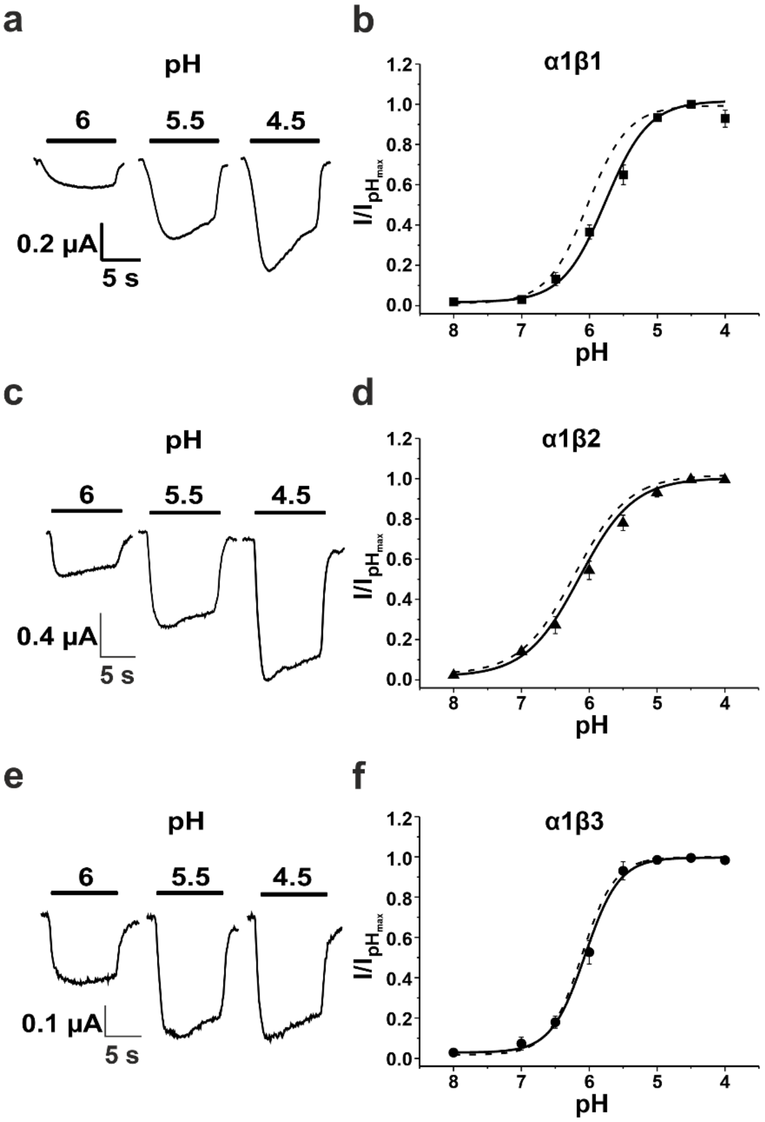

Supplementary Figure 3. Oocytes expressing subunits resulting from individual RNAs display proton induced currents. Proton-induced chloride currents in oocytes expressing heteromeric $GABA_A$ receptors composed of α1β1 (**a**), α1β2 (**c**) and α1β3 subunits (rat) (**e**) with corresponding pH-response curves in (**b, d, f**) with respective pH_{50} values: pH_{50} = 5.76 ± 0.04, n_H =1.36 ± 0.11 for α1β1, pH_{50} = 6.14 ± 0.09, n_H =1.09 ± 0.14 for α1β2 and pH_{50} = 6.06 ± 0.03, n_H =1.73 ± 0.14 for α1β3. The data are presented as mean values ±SEM, n= 5. Superimposed dotted lines illustrate pH-response curves of the corresponding homomeric receptors from Fig. 1.

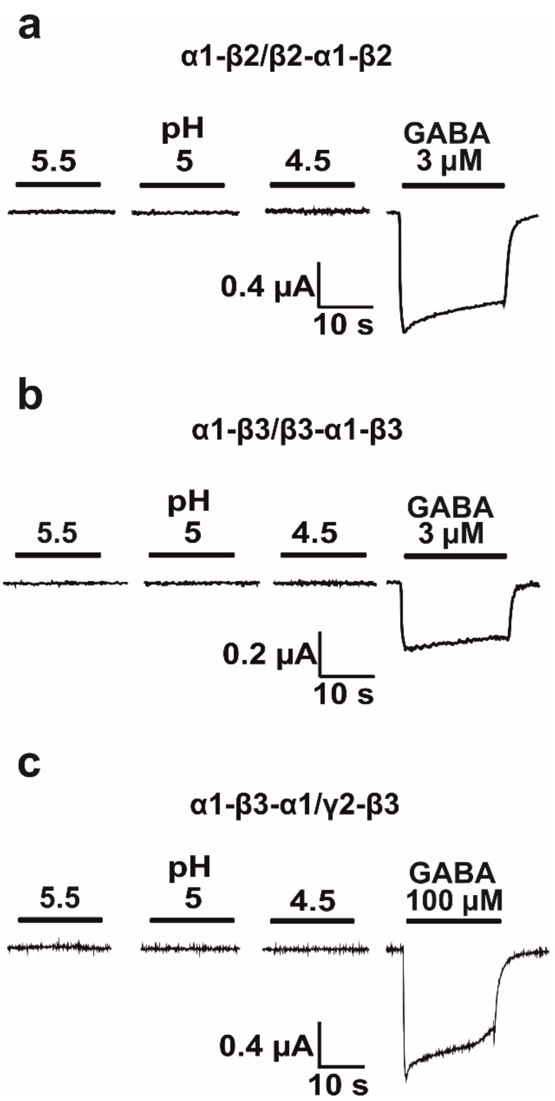

Supplementary Figure 4. Concatenation of subunits prevents activation of $I_{H(\beta)}$. GABA$_A$ receptors composed of concatenated subunits α1-β2/β2-α1-β2 (rat) (**a**), α1-β3/β3-α1-β3 (rat) (**b**) and α1-β3-α1/γ2-β3 (rat) (**c**) are not activated by changing the pH from 9 to indicated values (**a**, **b** and **c,** respectively) but activated by GABA (pH 7.2).

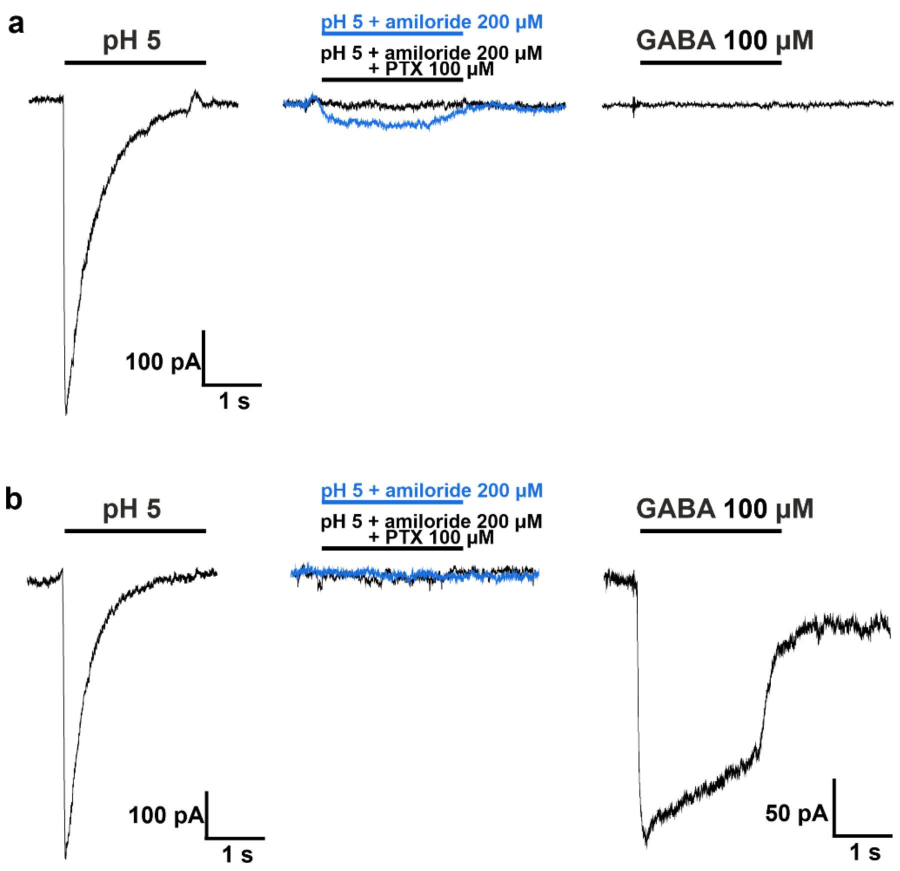

Supplementary Figure 5. Proton- and GABA-induced currents in Jurkat cells (a) and iPS cell-derived iCell GABANeurons (b).

a Representative currents recorded from Jurkat cells upon shifting the pH from 7.2 to 5 alone (left) or in the presence of 200 µM amiloride (middle overlaid currents, blue). Application of pH 5 produced ASIC-like fast activating currents, whereas co-application of pH 5 with amiloride (an ASIC blocker[2]) resulted in slower activating and desensitising currents that were completely blocked by picrotoxin (100 µM). Application of GABA did not activated I_{GABA} in these cells. Average mean current amplitudes are 440 ± 118 pA for pH 5-induced currents and 34 ± 4 pA for pH 5 in presence of 200 µM amiloride (n = 11, the data are presented as mean values ±SEM).

b Representative currents recorded from iPS cell-derived iCell GABAergic neurons cells. Application of pH 5 resulted in a current (left) which was completely abolished by co-application with amiloride (middle trace), consistent with an ASIC current. I_{GABA} (induced by 100 µM GABA pH 7.2) is shown on the right (indicating the presence of heteromeric $GABA_A$ receptors in these neurons). Average current amplitudes are 225 ± 44 pA for pH 5-induced ASIC currents and 308 ± 35 pA for GABA-induced currents (n = 5, the data are presented as mean values ±SEM).

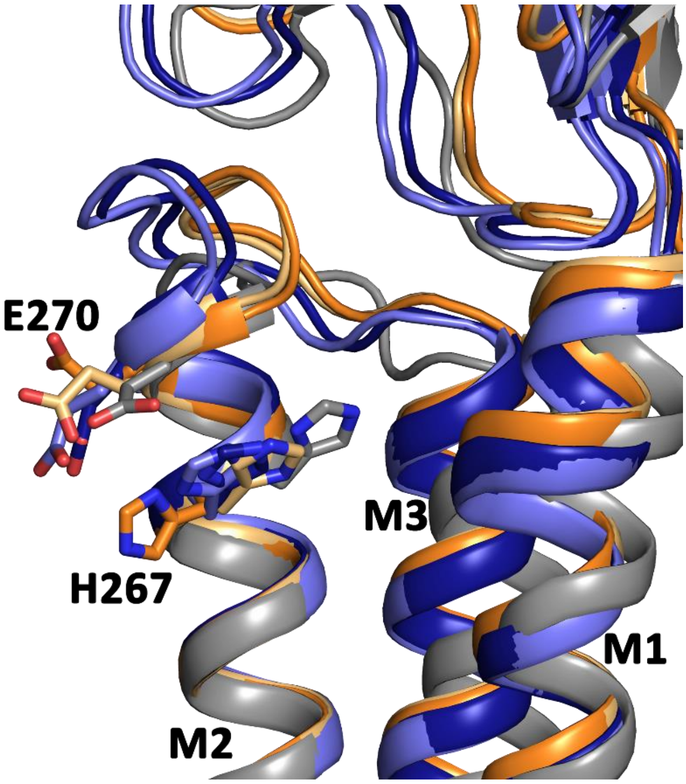

Supplementary Figure 6. Alignment of the M2 segment of different experimental structures of beta subunits. The protein is represented as cartoon; residues H267 and E270 (numbering corresponds to β3 numbers) are shown as sticks. The β3 subunits are colored in slate blue (4COF[3]) and dark blue (7A5V[4]), β1 subunit in grey (6DW0[5]) and the β2 subunits of the cryo-EM structure 6X3T[6] in light orange (chain A) and dark orange (chain E). Positions of H267 and E270 are equivalent and only slight differences in the rotameric state of are visible. We therefore expect these residues play a structurally equivalent role in homopentamers of β1 and β2. In the heteropentamers, the TMD adopts a different conformation, which limits this structural comparison due to the lacking structure of homomeric β1 or β2 structures.

Supplementary references

1. Sigel, E., Baur, R., Malherbe, P. & Möhler, H. The rat β1 -subunit of the GABAA receptor forms a picrotoxin-sensitive anion channel open in the absence of GABA. *FEBS Letters* **257**, 377–379 (1989).

2. Waldmann, R., Champigny, G., Bassilana, F., Heurteaux, C. & Lazdunski, M. A proton-gated cation channel involved in acid-sensing. *Nature* **386**, 173–177 (1997).

3. Miller, P. S. & Aricescu, A. R. Crystal structure of a human GABAA receptor. *Nature* **512**, 270–275 (2014).

4. Nakane, T. *et al.* Single-particle cryo-EM at atomic resolution. *Nature* **587**, 152–156 (2020).

5. Phulera, S. *et al.* Cryo-EM structure of the benzodiazepine-sensitive α1β1γ2S tri-heteromeric GABAA receptor in complex with GABA. *Elife* **7**, (2018).

6. Kim, J. J. *et al.* Shared structural mechanisms of general anaesthetics and benzodiazepines. *Nature* **585**, 303–308 (2020).
