# Supplementary material for: β subunits of GABAA receptors form proton-gated chloride channels: Insights into the molecular basis
Source: Commun Biol. 2022 Aug 3;5:784. doi: 10.1038/s42003-022-03720-2 (PMC9349252; doi:10.1038/s42003-022-03720-2)
Supplement: Supplementary file 3 — Description of Additional Supplementary Files [file 42003_2022_3720_MOESM3_ESM.pdf]

Description of Additional Supplementary Files

File name: Supplementary Data 1
Description: Source data underlying the figures.
