# Supplementary material for: β subunits of GABAA receptors form proton-gated chloride channels: Insights into the molecular basis
Source: Commun Biol. 2022 Aug 3;5:784. doi: 10.1038/s42003-022-03720-2 (PMC9349252; doi:10.1038/s42003-022-03720-2)
Supplement: Supplementary file 5 — Reporting Summary [file 42003_2022_3720_MOESM5_ESM.pdf]

Corresponding author(s): Aleksandra Garifulina, Steffen Hering

Last updated by author(s): May 9, 2022

Reporting Summary

Nature Research wishes to improve the reproducibility of the work that we publish. This form provides structure for consistency and transparency in reporting. For further information on Nature Research policies, see our Editorial Policies and the Editorial Policy Checklist.

Statistics

For all statistical analyses, confirm that the following items are present in the figure legend, table legend, main text, or Methods section.

n/a	Confirmed	
☐	☒	The exact sample size (n) for each experimental group/condition, given as a discrete number and unit of measurement
☐	☒	A statement on whether measurements were taken from distinct samples or whether the same sample was measured repeatedly
☐	☒	The statistical test(s) used AND whether they are one- or two-sided<br>*Only common tests should be described solely by name; describe more complex techniques in the Methods section.*
☒	☐	A description of all covariates tested
☒	☐	A description of any assumptions or corrections, such as tests of normality and adjustment for multiple comparisons
☐	☒	A full description of the statistical parameters including central tendency (e.g. means) or other basic estimates (e.g. regression coefficient) AND variation (e.g. standard deviation) or associated estimates of uncertainty (e.g. confidence intervals)
☐	☒	For null hypothesis testing, the test statistic (e.g. F, t, r) with confidence intervals, effect sizes, degrees of freedom and P value noted<br>*Give P values as exact values whenever suitable.*
☒	☐	For Bayesian analysis, information on the choice of priors and Markov chain Monte Carlo settings
☒	☐	For hierarchical and complex designs, identification of the appropriate level for tests and full reporting of outcomes
☒	☐	Estimates of effect sizes (e.g. Cohen's d, Pearson's r), indicating how they were calculated

Our web collection on statistics for biologists contains articles on many of the points above.

Software and code

Policy information about availability of computer code

Data collection	For two-microelectrode voltage-clamp recordings: Clampex Ver 10.1<br>For planar patch clamp recordings: PatchControl 384 (NANION)<br>Gromacs ver 5.1.2 (for Molecular Dynamics simulations)
Data analysis	For analysing electrophysiological data: Clampfit Ver 10.1 (two-microelectrode voltage-clamp data) and DataControl 384 (NANION) (planar patch clamp data)<br>Origin Software (OriginLab Corporation, USA) was used for generation of concentration–response curves and statistical analysis<br>For Molecular Dynamics simulations: Gromacs Ver 5.1.2 analysis tools, in-house Python scripts for graphs generation, PyMOL, VMD, CHAP computational tool

For manuscripts utilizing custom algorithms or software that are central to the research but not yet described in published literature, software must be made available to editors and reviewers. We strongly encourage code deposition in a community repository (e.g. GitHub). See the Nature Research guidelines for submitting code & software for further information.

Data

Policy information about availability of data

All manuscripts must include a data availability statement. This statement should provide the following information, where applicable:

- Accession codes, unique identifiers, or web links for publicly available datasets
- A list of figures that have associated raw data
- A description of any restrictions on data availability

Data supporting the findings of this manuscript are available from the corresponding authors upon reasonable request

Field-specific reporting

Please select the one below that is the best fit for your research. If you are not sure, read the appropriate sections before making your selection.

☒ Life sciences ☐ Behavioural & social sciences ☐ Ecological, evolutionary & environmental sciences

For a reference copy of the document with all sections, see nature.com/documents/nr-reporting-summary-flat.pdf

Life sciences study design

All studies must disclose on these points even when the disclosure is negative.

Sample size	The sample size has been determined and optimised on the basis of previous studies
Data exclusions	No data were excluded from the study
Replication	All data obtained were independently reproducible 4 or more times. the exact number of replications of each experiment is indicated in the figure captions.
Randomization	Randomization is not relevant to this study
Blinding	Investigators were not blinded

Reporting for specific materials, systems and methods

We require information from authors about some types of materials, experimental systems and methods used in many studies. Here, indicate whether each material, system or method listed is relevant to your study. If you are not sure if a list item applies to your research, read the appropriate section before selecting a response.

Materials & experimental systems

n/a	Involved in the study	
☒	☐	Antibodies
☐	☒	Eukaryotic cell lines
☒	☐	Palaeontology and archaeology
☐	☒	Animals and other organisms
☒	☐	Human research participants
☒	☐	Clinical data
☒	☐	Dual use research of concern

Methods

n/a	Involved in the study	
☒	☐	ChIP-seq
☒	☐	Flow cytometry
☒	☐	MRI-based neuroimaging

Eukaryotic cell lines

Policy information about cell lines

Cell line source(s)	ATCC, Sigma Aldrich, Fujifilm CDI
Authentication	ATCC authentication and routine visual inspection of cell morphology
Mycoplasma contamination	The cell line tested negative for mycoplasma contamination
Commonly misidentified lines (See ICLAC register)	No commonly misidentified lines were used in this study

Animals and other organisms

Policy information about studies involving animals; ARRIVE guidelines recommended for reporting animal research

Laboratory animals	The oocytes from Xenopus laevis frogs (NASCO (Fort Atkinson, USA)) were used for the heterologous expression of GABAA receptors
Wild animals	No wild animals were involved in this study
Field-collected samples	The study did not involve samples collected from the field
Ethics oversight	All experiments involving animals were approved by the Austrian Animal Experimentation Ethics Board in compliance with the

Ethics oversight	European convention for the protection of vertebrate animals used for experimental and other scientific purposes ETS no.: 123, which is in line with the EU Directive 2010/63/EU (GZ BMBWF-66.006/0021-V/3b/2019)

Note that full information on the approval of the study protocol must also be provided in the manuscript.
